# Monitoring HIV DNA and cellular activation markers in HIV-infected humanized mice under cART

**DOI:** 10.1186/s12985-018-1101-9

**Published:** 2018-12-17

**Authors:** Mary-Aude Rochat, Erika Schlaepfer, Stefan P. Kuster, Duo Li, Annette Audige, Sandra Ivic, Audrey Fahrny, Roberto F. Speck

**Affiliations:** Department of Infectious Diseases and Hospital Epidemiology, University Hospital of Zurich, University of Zurich, Raemistrasse 100, 8091 Zurich, Switzerland

**Keywords:** HIV-1, HIV reservoir size, Humanized mice, cART, Alu-PCR

## Abstract

**Background:**

The major obstacle to cure of HIV type-1 infection is the presence of the HIV reservoir, hidden from the immune system and insensitive to combined antiretroviral therapy (cART). Eradication approaches have been hindered by the difficulty for accurately monitoring its size in vivo, especially in the lymphoid organs. Humanized mouse models are a valuable tool for systematically assess the efficacy of therapeutic interventions in reducing the HIV reservoir. Nonetheless, persistence of the HIV reservoir over time, in the presence of cART, has yet to be analyzed in this in vivo model.

**Findings:**

We found that the proviral DNA as well as the total DNA were very stable in the spleen and mesenteric lymph node irrespective of the length of cART. Notably, the amount of proviral DNA was very similar in the spleen and lymph node. Furthermore, we observed a correlation between the percentage of splenic human CD4+ T-cells with total HIV DNA, between the number of human CD38 + CD8+ T-cells in the spleen with the amount of integrated HIV DNA, and eventually between the hCD4/hCD8 ratio in the spleen with integrated as well as total HIV DNA implying that the CD8+ T cells influence the size of the HIV reservoir.

**Conclusions:**

Here, we demonstrated the stability of this reservoir in humanized mice irrespective of the length of cART, confirming the relevancy of this model for HIV latency eradication investigations. Notably, we also found correlates between the frequency of CD4+ T-cells, their activation status and viral parameters, which were analogous to the ones in HIV-infected patients. Thus, hu-mice represent a very valuable HIV latency model.

## Introduction

The cure for HIV is impeded by the latent reservoir of HIV, which is established during acute HIV infection, is non-responsive to cART [1, 2] and has a very slow decay rate with a ~t½ of 44 months [3, 4]. Memory CD4+ T-cells with ~1/10e5 latently infected [5] are the major cell subset making up this latent reservoir [6]. The persistence of this reservoir might be mediated by either cellular intrinsic longevity [7] and self-renewal capacity [8] or homeostatic proliferation through cytokines and cell-cell interactions [6, 9].

Several humanized mouse (hu-mice) models recapitulate key features of HIV infection [10–13] and have been successfully used for studying various eradication approaches [14–17]. However, this model has yet to be validated for some key aspects of HIV latency such as for its stability over time. Furthermore, hu-mice are chimeric animals, where the human hematopoietic turnover might be altered [18]. Partial interspecies cross-reactivity between murine cytokines/chemokines or MHC and co-stimulatory molecules with their human cognate receptors, might reduce the survival capacity of engrafted human cells, possibly affecting the decay of the latent reservoir.

Here we explored the intricate network between the HIV reservoir and its potential impact on HIV pathogenesis in HIV-infected hu-mice. In particular, we quantified the HIV DNA in hu-mice treated for different length with cART and explored whether it correlated with the viral load before cART, the number of target cells and immune activation markers. We will explicitly use the term HIV reservoir as measuring HIV DNA will capture defective as well as infectious HIV particles [19]. The term latent reservoir is primarily reserved for latently infected cells which retain the capacity to produce infectious virus particles [20] and requires assays measuring virus particle production or transcribed HIV mRNA following activation of latently HIV-infected cells [19].

## Materials and methods

### Generation of humanized mice

Immunodeficient NOD-scid IL2Rgammanull (NSG) mice were obtained from the Jackson laboratory (#005557) and bred in a specific pathogen free (SPF) animal facility (Ito et al. 2002). Newborn mice were sublethally irradiated (1Gy) within 5 days of birth and 2e10^**5**^human cord blood-derived CD34+ cells were injected intra-hepatically. These CD34+ cells were isolated using immunomagnetic beads (Miltenyi, #130–046-702) after gradient centrifugation (Axis-Shield PoC AS, Norway). The purity (> 90%) of the CD34+ was determined by flow-cytometry. 12 weeks after human HSPCs’ transplantation, human reconstitution in the blood was analyzed by flow-cytometry with the following markers; hCD45, hCD19, hCD3, hCD4, hCD8. Animals with more than 10% of human reconstitution were selected for HIV infection.

### HIV infection

293 T cells, obtained from ATCC® CRL-3216™, were cultured in DMEM medium (Sigma, D6429), supplemented with 10% of FBS (Millipore, S0615) and 1% penicillin/streptomycin (Gibco, #15140–122) and transfected with 20 μg of lab-strain Yu2 plasmid DNA, using Polyethylenimine (PEI, 2 μg/μg of plasmid, Polyscience #23966). After 48 h, the medium was collected, 0.22 μm-filtered (Steriflip, Millipore, SCGP00525) and stored at − 80 °C until use. The 50% of tissue culture infectious dose (TCID50) was determined by incubating 1:5 serial dilution of the HIV virus stock, in quadruplicate, with 2*10^**5**^ activated PBMCs for 7 days. TCID50 was estimated based on the number of p24-positive wells and the Reed and Muench calculation. Isoflurane-anesthetized animals were intra-peritoneally injected with HIV Yu2 (2*10^**5**^ TCID50). Four weeks later, viral dissemination was determined using the Cobas® Amplicor technology (Roche). Upon viral replication, animals were fed with cART-supplemented food pellet until euthanasia. Confirmation of undetectable HIV RNA copies/ml (limit of detection of 440 copies/ml) was determined after 5 weeks post cART initiation. Animals were euthanized at viral dissemination (week − 1), at viral suppression (week 5) and after 6, 9, 13 and 17 weeks of cART.

### cART-supplemented food pellet

Production of cART-supplemented food pellet was adapted [10]. Briefly, 4700 mg/kg of Raltegravir (Isentress, MSD Merck Sharp & Dohme, AG), 540 mg/kg of Tenofovir disoprexil (TDF, Viread, Gilead Science Switzerland Sàrl) and 540 mg/kg of 3TC (Lamivudin, ViiV, Healthcare GmbH) were mixed with ground protein-rich, vitamin-fortified food (Nafag 3432, Provimi Kliba AG, Switzerland), subsequently gamma-irradiated with 25 kGy.

### Peripheral blood and tissues collection

**Fig. 1:**
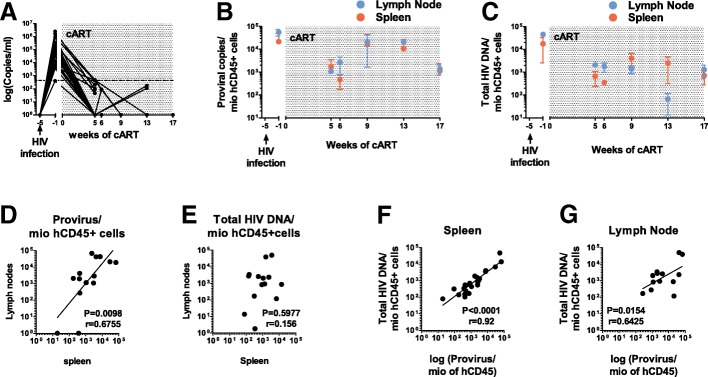
Stability of the HIV reservoir in humanized mice in the spleen and lymph node. Infected NSG mice (*n* = 36) were euthanized prior to cART (*n* = 3) and after documented viral suppression at 5 (*n* = 2), 6 (*n* = 10), 9 (*n* = 9), 13 (*n* = 7) and 17 (*n* = 5) weeks of treatment. **a** Viral load in hu-mice over time before and after treatment with cART. Spleen and lymph nodes were removed at euthanasia and HIV provirus (**b**) and total HIV DNA/millions of hCD45+ cells (**c**) quantified over time shown in blue for the lymph node and in red for the spleen (mean ± SEM). **d** Correlation between proviruses/millions of hCD45+ cells between the spleen and the lymph node from the same animal or pool of animals. **e** Correlation between total HIV DNA between the lymph node and the spleen from the same animal or pool of animals, (*P* = 0.2089, *r* = 0.311). **f** and **g** Correlation between proviruses and total HIV DNA in the spleen or the lymph node, respectively. Because of the low yield of lymphatic tissues in some mice, we were forced to pool the lymphatic tissues of some mice, which had a similar peak viral load. In fact in some mice we were not able to retrieve lymph node tissue. Thus, the data presented for the lymph nodes are based on 26 mice euthanized and eventually 14 (**b**) and 16 (**c**) data points, and for the spleen on 36 mice with 24 data points

Blood was collected retro-orbitally in an EDTA-coated tube (BD microtainer K2E, #365975), after 4 weeks of HIV infection, 5 weeks of cART-supplemented food initiation and at the euthanasia. Separation of the plasma and the cell pellet was obtained by low speed centrifugation (3′000 rpm for 10 min). Organs were meshed in MACS buffer (PBS supplemented with 2% FBS and 2 mM of EDTA (Invitrogen, #15575–038) using a 70 μm strainer (Falcon 352,350) and the syringe plug (B/braun Injekt, 10 ml, #4606108). Splenocytes were separated from the erythrocytes by gradient centrifugation, and the lymph nodes were lysed with 1 ml of ACK buffer (Gibco, A1049201). PBS was used for the washing steps. Cell suspensions from organs were counted, stained for flow-cytometry analysis and frozen as dry pellet for the PCR analysis. The pool of some specimens with low cell numbers was performed according to the time of suppression and the baseline viral load. We euthanized mice prior and 5, 6, 9, 13 and 17 weeks post-cART (see legend to Fig. 1 for the number of mice euthanized).

### Antibodies and reagents

Humanized mice-derived samples were analyzed for their human cell distribution by flow-cytometry using the following antibodies from Biolegend: CD19-APC (#302212), CD3-PE (#300308), CD4-PECy7 (#300512), CD8-Brilliant Violet 421 (#301036). CD38-PerCPCy5.5 (#303521) and HLA-DR-FITC (#307604). The pan-human marker CD45-Krome Orange, was obtained from Beckman Coulter (PN96416).

### Flow cytometry

Blood and organs collected from animals were incubated with the cell-surface marker antibody at an optimized dilution in FACS buffer (PBS containing 2 mM EDTA, 0.1% sodium azide and 10% FCS) for 20 min at 4 °C. After staining, blood samples were lysed with 1x BD FACS™ Lysing solution (BD, #349202) for 10 min at room temperature, subsequently washed with PBS, whereas cell suspension of organs were washed with FACS buffer. Stained cells were fixed with 1% paraformaldehyde (PFA) in PBS, acquired on a CyAn TM ADP Analyzer (Beckman Coulter) and data were analyzed using FlowJo (version V.10.0.8). We defined the live cells by the side scatter/forward scatter gate and then quantified the number of cells by the specific marker of interest.

### DNA extraction and quantitative real-time PCR for total HIV gag

DNA was extracted from organs using the QIAmp DNA blood minikit (Qiagen, #51106), following manufacturer’s instructions. DNA elution was performed using 100 μl of 10 mM Tris-HCl at pH 8 (Affymetrix, #1185-53-1). The eluate was used for a second round of elution to maximize the DNA recovery. DNA concentration was quantified using the Quant-iTTM PicoGreen® dsDNA Assay (Thermofisher Scientific, P11496). Subsequently, total HIV DNA copies and human cell quantification was performed using 100 ng of DNA per replicate. Amplification of gag sequence was performed using the following primers-probe; 1 μM of Forward: 5-CAAGCAGCCATGCAAATGTTAAAAGA-3; 1 μM of Reverse: 5-TACTAGTAGTTCCTGCTATGTCACTTCC-3 and 350 nM of the probe: 5-FAM-TGCAGCTTCCTCATTGATGGT-BHQ1–3. Human cell quantification, within a mouse background, relayed on divergence in the RPP38 gene sequence [21], which is detected using 400 nM of primers (Forward: 5-TCACGACACCTCTGCTTTA-3 and Reverse: 5-AGCGGTGAGAAACTAGGAA-3) with 100 nM of probe (5-FAM-AAGTTGCTTCACACTGGAACGCTTGC-BHQ1–3). The PCR mix contained 2x Maxima Hot Start (ThermoFischer Scientific, K1052), Molecular grade water (Applichem, A7398–0500) and Rox (dilution 1/100, ThermoFischer Scientific, #12223012), as a reference dye. PCRs were run on an ABI Prism 7500 cycler with these cycling conditions: 5 min at 95 °C followed by 50 cycles of 5 s at 95 °C and 30 s at 60 °C, where the annealing, elongation and signal detection occur. Standard curves for the copy number determination were prepared using different ratios of cell lines, which DNA was independently extracted. In brief, increasing concentration of either 8E5 (NIH, cat. 95, 2 copies/genomes) into Jurkat T cells (ATCC® TIB152TM), for the gag standard curve or Jurkat T cells into EL4 (mouse leukemic cell line ATCC® TIB39TM), for the human standard curve, were used at 100 ng per replicate, which approximate 15′000 human cells or 30′000 gag copy number.

### Alu-PCR

Determination of integrated provirus DNA was adapted from previously described methods by Spiegelaere et al. 2014. In brief, 50 ng of DNA per replicate (96 wells plate, Starlab, I1402–9700) was amplified with either *alu* (100 nM, 5-GCGCGGTGGCTCACGCCTGTAAT-3) and *gag* (600 nM, 5-CTTAATACTGACGCTCTCGCACC-3) or *gag* only primers in a final volume of 50 μl. The cycling conditions are the following: 5 s at 95 °C, 40 cycles of 45 s at 95 °C, 60 s at 58 °C and 210 s at 72 °C with a final elongation of 10 min at 72 °C. The repetitive sampling was performed with 42 replicates and the amount of DNA depended on the total HIV copy number determined previously. Then, 10 μl per well is transferred into 10 μl of the nested PCR mix, without water, for the amplification of LTR sequence. The Forward (5-ATAAAGCTTGCCTTGAGTG-3) and the reverse (5-TGACTAAAAGGGTCTGAGGGATCTCTA GTTACCAG-3) primers were used at 1 μM and the LNA probe (Eurogentec, 5-FAM-TG-lnT-G-lnT-GC-lnC-C-lnG-T-BHQ1–3) at 300 nM. The PCR conditions were as followed: 95 °C for 5 min; 50 cycles at 95 °C for 10 s, 55 °C for 5 s and 60 °C for 40 s with a final elongation of 10 min at 72 °C. Alu-gag and gag only CT values were transferred into the excel sheet from Spiegelaere with the amount of human cells per well, determined using the human quantification PCR described above. In addition, the estimation of integrated proviruses required the assessment of a PCR-intrinsic error, which has been calculated using a standard curve of J-lat clone 9.2 (NIH, #9848) and 15.4 (NIH, #9850), 8E5 as well as ACH2 cells (NIH, #349) cultured with Efavirenz (1 μM, Sigma, SML0536) and AZT (5 μM, Sigma, A2169) with a Jurkat T cell background. The error obtained from the standard 10/10^**6**^, 100/10^**6**^ and 10^**3**^/10^**6**^ (standard deviation of 0.028) was 0.4516.

### Statistics

The software GraphPad Prism Version 5.04 was used for doing statistics. For comparing total HIV DNA or proviral DNA prior vs after cART, we used the Mann-Whitney test. Stability of integrated proviruses or total HIV DNA over time was determined by ANOVA and linear regression between all the data sets. For investigating correlations between the various parameters, we used the Spearman’s rank-order correlation test.

## Results and discussion

In humans, many factors are implicated in the size of the HIV DNA reservoir [22]. Among others, it has been shown that the pool of latently infected cells continuously expands at variable rates in cART naïve HIV-infected patients, depending on the patient’s cytotoxic T lymphocytes (CTL) response [23, 24] and the immune activation [25]. Furthermore, the CD4+ T-cell count at the time of cART initiation correlated negatively with the total HIV DNA in the blood and in the gut [26]. Markers of immune activation were predictors of proviral DNA [27]. In cART treated patients, it was shown that HIV integrates preferentially at specific sites, favoring their proliferation [28, 29]. Thus, HIV DNA is not a stochastic process.

The generation of hu-mice has allowed effective HIV knowledge expansion and straightforward therapeutic approach evaluations [30]. However, comprehensive characterization of HIV latency is lacking. Indeed, parameters influencing the size of the HIV reservoir are unknown and likely numerous, i.e.*,* chimerism level, immune activation and cellular proliferation, sensitivity of the graft to HIV, infection dose, initial viral load and duration of HIV disease before cART initiation [31]. Exact quantification of the HIV reservoir is crucial for judging any intervention targeting the latent reservoir.

Here we explored the persistence of the HIV reservoir depending upon the length of cART and in the intricate context of HIV pathogenesis. Notably, latency is interrogated either by viral outgrowth assays (VOA) or by PCR-based methods [32, 33]. VOA detect solely the number of cells productively infected. In contrast, DNA PCR-based methods detect all HIV genomes irrespective whether they are fully infectious or defect. Defect proviruses may still produce viral antigens or replication-incompetent HIV that activates the immune system and thereby contributes to HIV pathogenesis. Thus, we interrogated here the dynamic of the HIV reservoir by quantifying total and proviral HIV DNA and by measuring a number of cellular markers.

Briefly, we infected hu-mice i.p. with the CCR5-tropic strain, YU-2 and started cART consistently 4 weeks later, since length of natural HIV infection is a factor influencing the size of the HIV reservoir. Notably, HIV dissemination occurred within 4 weeks following HIV infection, defined as baseline viral load, and HIV replication was suppressed within 5 weeks of cART (Fig. 1a).

We treated the mice up to 17 weeks (Fig. 1a). Notably, we noticed that total HIV DNA in the spleen prior to cART differed significantly from the ones under cART (avg ± sem of total HIV DNA/mio CD45+ cells prior (*n* = 3) vs after start of cART (*n* = 21): 17605 ± 15,042 vs 1639 ± 712, *p* = 0.036 (Mann Whitney test)) whereas this was not the case for integrated DNA (avg ± sem of proviral copies/mio CD45+ cells prior vs after: 29032 ± 16,762 vs 45,505 ± 3383; *p* = 0.0809). The rapid decay observed for total HIV DNA is most likely due to the decay of linear unintegrated HIV DNA [34] – integrated forms decay more slowly [35]. These data are consistent with observations obtained in HIV-infected humans [34, 35].

We then wondered whether the length of cART has any impact on the size of the HIV reservoir in the HIV-infected hu-mice. Once the viral load was suppressed, the proviral DNA as well as the total HIV DNA were very stable in the spleen and the mesenteric lymph node (mLN) and comparable over the entire 12 weeks observation period independent of the length hu-mice received cART (Fig. 1b and c). Notably, the value of total HIV DNA at week 13 in the mLN was unusual and we considered it as an outlier since the value at week 16 was superimposed again. In human, HIV DNA shows a bi-exponential decay phase [36] with a first half-life based on mathematical modelling of 113 days and a second half-life of years [37]. At least for the 12 weeks once HIV RNA was suppressed, (i.e., first documented suppression of viral load at week 5, total duration of cART 17 weeks), we did not see a decay of the HIV DNA. The discrepancy between decay rates in this HIV mouse latency model and humans may be inherent to the mouse model, the distinct lymphoid tissues examined (i.e., spleen and lymph nodes in mice vs PBMCs in human) or explained by the large inter-individual heterogeneity of HIV DNA seen in mice and patients [35], and the rather limited number of *n* = 33 mice used to look into this issue. Notably, the tiny amount of PBMCs in blood from humanized mice preclude this kind of analysis, and correspondingly we were not able to perform a direct comparison of identical lymphoid tissue specimens.

Importantly, the amount of proviruses in the spleen and the mLN from the same animal or pool of animals correlated significantly (Fig. 1d), indicating that identical pathogenic processes underlie the establishment of the HIV reservoir in the two lymphoid compartments. This correlation, between spleen and mLN, was not observed for total HIV DNA (Fig. 1e), pointing to distinct properties in these two lymphoid compartments as related to HIV DNA stability or/and cellular proliferation rate. As previously shown [6, 27, 38], we found a strong correlation between the integrated and the total HIV DNA per million of hCD45+ in the spleen as well as in the mLN (Fig. 1f and g). The similar amount of integrated (Fig. 1b) and total HIV DNA (Fig. 1c) during productive infection (at − 1 week of cART) could be due to different sensitivities of the PCR methods used. Therefore, the values obtained for both parameters cannot be directly compared; their relationship, however, remains valid for understanding the dynamics of both compartments. Similar to human [23], the size of the HIV reservoir was highly variable between the individual animals. Notably, the gut associated lymphoid tissues (GALT) is a major site of HIV replication early in HIV infection [39] and the mLN is crucial for the induction and the regulation of specific immune responses initiated in the gut [40]. The ability to study HIV latency in the mLN is a clear additional benefit of HIV-infected humanized mice. In particular, it enables to explore the penetrance and efficacy of latency reversing agents in lymphoid tissues, as the penetrance of ART has been shown to vary depending the lymphoid organ analyzed [41].

**Fig. 2:**
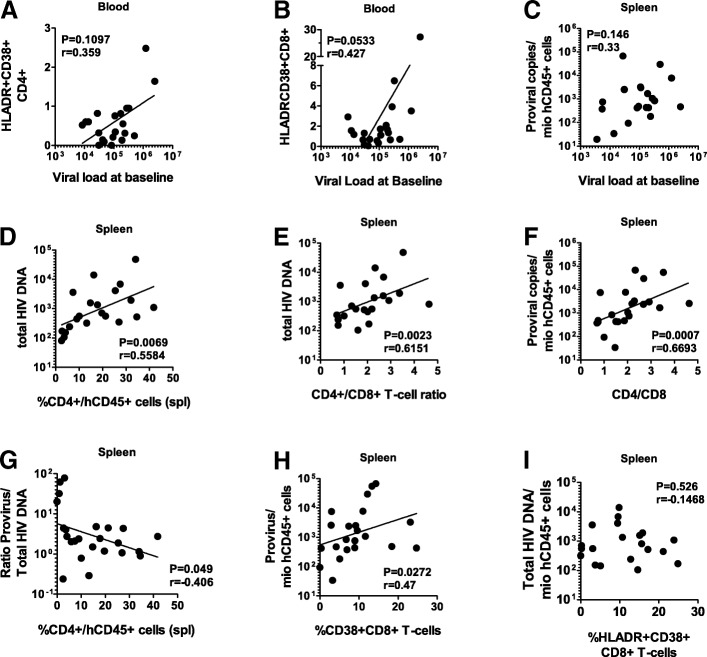
Association between the reservoir size, viral load, hCD4+ T cells and immune activation. **a**, **b** Correlation between the viral load and the percentage of HLADR+CD38 + hCD4+ (**a**) and hCD8+ T-cells (**b**) in the blood at 4 weeks p.i.. **c** Correlation between number of proviruses/millions of hCD45+ cells in the spleen with the baseline viral load (*P* = 0.4626, *r* = 0.157). **d** Correlation between total HIV DNA/millions of hCD45+ cells in the spleen with the blood frequency of CD4+ T cells at 4 weeks p.i.. **e** and **f** Correlation between proviruses and total HIV DNA with the CD4+/CD8+ T-cell ratio in the spleen. **g** Correlation between the ratio proviruses/total HIV DNA with the frequency of CD4+ T cells in the spleen. **h** Correlation between the number of proviruses/millions of hCD45+ cells with the percentage of CD38+ hCD8+ T cells in the spleen. **i** Correlation between the total HIV DNA/millions of hCD45+ cells with the frequency of HLADR+CD38+ hCD8+

Association between immune activation, especially activated hCD4+ and hCD8+ cells, and viral replication, in the absence of cART, has been widely reported in patients [25, 42]. Here, we found a tendency of correlation between the baseline viral load and the frequency of HLA-DR + CD38+ hCD4+ (Fig. 2a) and hCD8+ T cells (Fig. 2b) in hu-mice, recapitulating HIV features in HIV-infected individuals [42–44]. As reported [25], we could not find any correlation between the amount of proviruses under cART and baseline viral load (Fig. 2c) [45, 46]. Of note, the frequency of hCD45+ or hCD4+ T-cells at the time of HIV infection or after 4 weeks was neither associated with the baseline viral load nor with the amount of integrated HIV DNA (data not shown). These data refuted the chimerism level or the frequency of target cells, respectively, as relevant indicators of HIV viral load and size of the HIV reservoir in hu-mice.

We wondered whether the size of the HIV reservoir was dependent on the hCD4+ T-cells. Indeed, the percentage of splenic hCD4+ T cells correlated with total HIV DNA (Fig. 2d). There was a trend between the percentage of splenic hCD4 cells and the proviral DNA (*P* = 0.1318, *r* = 0.3165). Strikingly, we also observed a correlation between the hCD4/hCD8 ratio in the spleen with integrated as well as total HIV DNA (Fig. 2e and f) implying that the CD8+ T cells influence the size of the HIV reservoir.

The ratio of integrated/total HIV DNA correlated negatively with the frequency of hCD4+ T-cells in the spleen (Fig. 2g). These findings could be explained by high hCD4+ T-cell proliferation in response to low hCD4 prior to cART [6], thereby increasing the integrated/total HIV DNA ratio by diluting the total HIV DNA. Alternatively, mice with a higher percentage of CD4+ T-cells could have more cells prone to pre-integration latency, and that the number of cells resulting eventually in post-integration latency (proviral DNA) is not affected by the height of CD4+ T-cells. We did not observe such a correlation when looking at the total DNA in the mLN. Irrespective of differences in the lymphoid compartment studied, these data confirm CD4+ T-cells as prominent HIV reservoir in hu-mice. The persistence of HIV-infected CD4+ T-cells in hu-mice under cART is most likely maintained through endogenous murine IL-7 driven homeostatic proliferation [47].

We also found that the amount of integrated HIV DNA correlated with the number of CD38 + hCD8+ T-cells in the spleen (Fig. 2h). These data are reminiscent of recent findings obtained in humans, i.e., in the blood proviral DNA correlated with the frequency of HLA-DR+ CD8+ T-cells [27] and in the gut total HIV DNA with singly activated CD8+ T-cells (CD38+ or CD69+) [48, 49]. We did not observe any correlation between total HIV DNA and activated cells, including HLA-DR+ CD38+ CD8+ T-cells, implying that amount of total HIV DNA and cellular activation are subjected to distinct mechanism(s).

The data we present complement very nicely the data reported by Arainga et al. [50]. They identified the central memory T-cells as the dominant latently HIV infected T-cell in HIV-infected hu mice treated for 4 weeks with cART. Besides and very importantly, they found that mature macrophages harbored also proviral DNA.

In summary, we demonstrate that the HIV reservoir as quantified by HIV DNA in the spleen and lymph nodes is stable over a cART treatment period of 17 weeks. Rather all previous reports studied the effects of cART in HIV-infected hu mice for at most ≈6–8 weeks. In those previous studies, we and others showed the successful reversal of latency by either conventional or modified viral outgrowth assays or viral rebound after interruption of cART [10, 11, 51, 52]. Notably, and consistent with our data, Lavender et al. showed viral rebound after 18 weeks of cART mediated viral suppression [53]. We also report here that the HIV DNA correlates with immune activation, the number of HIV target cells present and that it is apparently affected by the CD8+ T-cell population. Notably, first studies were recently published examining “kick” and “kill” strategies in HIV-infected hu-mice [14, 54].

A large number of different background mouse strains are today available which upon transplantation of human CD34+ cells, present different functional immune cell activities, among others functional CD8+ and NK cells [55]. Thus, compounds which act indirectly on HIV-infected cells can also be explored for their effects on the HIV reservoir. Thus, humanized mice represent a very attractive in vivo HIV latency model [56].

## Abbreviations

cART: Combined anti-retroviral treatment
HIV: Human immunodeficiency virus (HIV) type 1
HSPCs: Hematopoietic stem and progenitor cells
hu-mice: Humanized mice
mLN: Mesenteric lymph node
